# Age-Related Gene Expression Differences in Monocytes from Human Neonates, Young Adults, and Older Adults

**DOI:** 10.1371/journal.pone.0132061

**Published:** 2015-07-06

**Authors:** Michelle M. Lissner, Brandon J. Thomas, Kathleen Wee, Ann-Jay Tong, Tobias R. Kollmann, Stephen T. Smale

**Affiliations:** 1 Department of Microbiology, Immunology, and Molecular Genetics, University of California Los Angeles, Los Angeles, California, United States of America; 2 Division of Infectious and Immunological Diseases, Department of Pediatrics, University of British Columbia, Vancouver, British Columbia, Canada; University of Alabama at Birmingham, UNITED STATES

## Abstract

A variety of age-related differences in the innate and adaptive immune systems have been proposed to contribute to the increased susceptibility to infection of human neonates and older adults. The emergence of RNA sequencing (RNA-seq) provides an opportunity to obtain an unbiased, comprehensive, and quantitative view of gene expression differences in defined cell types from different age groups. An examination of *ex vivo* human monocyte responses to lipopolysaccharide stimulation or *Listeria monocytogenes* infection by RNA-seq revealed extensive similarities between neonates, young adults, and older adults, with an unexpectedly small number of genes exhibiting statistically significant age-dependent differences. By examining the differentially induced genes in the context of transcription factor binding motifs and RNA-seq data sets from mutant mouse strains, a previously described deficiency in interferon response factor-3 activity could be implicated in most of the differences between newborns and young adults. Contrary to these observations, older adults exhibited elevated expression of inflammatory genes at baseline, yet the responses following stimulation correlated more closely with those observed in younger adults. Notably, major differences in the expression of constitutively expressed genes were not observed, suggesting that the age-related differences are driven by environmental influences rather than cell-autonomous differences in monocyte development.

## Introduction

Age-related differences in clinical susceptibility to infection have been extensively documented, with diminished protective responses and enhanced susceptibility observed in pre-term and term infants, as well as in older adults when compared to young adults [1–5]. This clinical observation of an age-dependent risk for infectious morbidity and mortality has led to an interest in identifying the underlying mechanisms and deriving strategies to enhance protective immune responses at the extreme ends of life [1–3].

Differences in innate immune responses are thought to contribute to the overall susceptibility observed in neonates and older adults [2,6]. Neonates have been reported to produce lower levels of effector molecules, such as oxygen radicals [2,7]. A number of other proteins have also been reported at reduced levels in innate immune cells, including reduced expression of IFNα, CD40, CD80, CD83, and CD86 in neonatal plasmacytoid dendritic cells [5]. Furthermore, newborns and older adults produce altered levels of cytokines that regulate the development of adaptive immunity (reviewed in [2]). For example, the heterodimeric, Th1-inducing innate cytokine, interleukin(IL)-12, is expressed at reduced levels in neonates, due to the reduced expression of its p35 subunit [8–10]. In contrast, the anti-inflammatory cytokine, IL-10, and the Th17-inducing cytokines, IL-6 and IL-23, have been observed at elevated levels in neonates [9,11]. In older adults, a variety of innate effector responses appear to be reduced, including superoxide generation and the phagocytosis of microorganisms [12,13]. Systemic low-level inflammation is another common characteristic of older adults that may alter their response to infection (reviewed in [2]).

The approaches used to identify age-dependent differences that lead to an increased risk to suffer from infection at the extreme ends of life have been largely balkanized and focused on a few particular components, the choice of which appears to depend on the expertise of a given group of investigators. What has been missing is an unbiased yet comprehensive interrogation of the events that occur in the very young and the very old following recognition of an infectious threat. In addition to our deficiency in knowledge of age-dependent differences in the immune system, little is known about the molecular mechanisms responsible for these differences. Reduced activation of transcription factors such as interferon response factor 3 (IRF3), defects in nucleosome remodeling, and differences in the expression of pattern recognition receptors and signaling molecules (e.g. MyD88) are among the mechanisms that have been proposed to be responsible for the diminished innate immune responses observed in neonates [2,14–16].

Age-dependent differences in hematopoietic stem cells and in the development of hematopoietic lineages have also been observed, providing one possible explanation for the immune response differences [17–19]. According to this scenario, myeloid cell types may be fundamentally different in neonates, adults, and older adults, resulting in different gene expression responses following stimulation or infection. As an alternative, the myeloid cell populations may be similar, but age-related differences in the blood or tissue microenvironment may lead to different responses [20]. The response differences may be lost when cells from different age groups are cultured under the same conditions, or they may be retained via epigenetic mechanisms or other memory mechanisms [3].

DNA microarrays were previously used to obtain genome-scale insight into age-dependent differences in gene expression following infectious exposure [15]. More recently, RNA sequencing (RNA-seq) has emerged as a more quantitative method for examining transcriptomes [21]. The availability of the RNA-seq method provides an opportunity to unravel, with greater precision, the age-dependent differences in the immune system that increase risk for a serious outcome following infection. As a first step, the identification of age-related differences in gene expression following *ex vivo* infectious exposure of defined cell populations, along with the identification of differences in constitutive gene expression in these populations, would be of considerable value.

In this study, RNA-seq was used to compare the gene expression responses to LPS stimulation or *Listeria monocytogenes* (*Lm*) infection in cord blood monocytes and in peripheral blood monocytes from young and older adults. LPS provides an example of a well-defined innate immune stimulator; *Lm* causes suffering and dying in the very old and the very young, while most young adults rarely even display symptoms if infected [22]. Our data reveal extensive similarities in constitutive gene expression and in the response to stimulation or infection in monocytes from the three age groups. Furthermore, most of the differences identified between neonates and young adults could be connected to the previously reported reduction in IRF3 activity in neonates [15]. In contrast, most differences between young adults and older adults appeared to result from a low-level inflammatory state (‘inflammaging’) that characterized monocytes from older adults. Interestingly, large differences in the expression of constitutively expressed genes, which would be expected if blood monocytes from neonates, adults, and older adults were fundamentally different, were not identified. This finding supports a hypothesis in which age-related environmental differences are responsible for the inability of neonatal monocytes to mount a robust IRF3-mediated response.

## Materials and Methods

### Isolation of cells and stimulation conditions

This study was specifically approved by the Research Ethics Board of the University of British Columbia (Protocol H13-00347). Informed written consent from all enrolled adult participants or the next of kin, care givers, or guardians on the behalf of the cord blood participants involved in our study was obtained for all study participants. Animal research for this study was specifically approved by the UCLA Chancellor’s Animal Research Committee (Protocol 1999-073-53E).

Samples of cord blood from healthy, full-term elective Caesarean sections without labor and samples of healthy young adult (ages 19–45) and older adult (aged 65 and older) peripheral blood were collected directly into sodium heparin-containing vacutainers (BD Biosciences). Mononuclear cells were isolated by density gradient centrifugation within two hours of blood collection to avoid alterations of cell properties [11]. Positive selection of monocytes from mononuclear cells was then carried out using Miltenyi microbeads according to the manufacturer’s protocol with some revisions. Briefly, mononuclear cells were incubated with 800 uL MACS buffer and 200 uL anti-human CD14 microbeads at 4°C. Cells were then washed with MACS buffer prior to positive selection of monocytes using Miltenyi selection columns. Purified monocytes from each donor were cultured in RPMI 1640 medium supplemented with Glutamax (Gibco, Life Technologies) and 10% human AB serum (Gemini Bio Products). The monocytes were counted and plated onto 96 well plates at a density of 1x10^6^ cells/well. Monocytes were immediately stimulated with LPS (10 ng/ml) (InvivoGen tlrl-eblps) for 0, 1, and 6 hrs, or were infected with *Lm* at MOI = 5 for 0, 2, and 6 hrs. These time points were selected on the basis of pilot experiments, which showed that they capture the first and second major waves of gene activation in response to LPS stimulation and *Lm* infection. Wild-type (WT) *Lm* strain 10403s was provided by Dr. D. Portnoy (University of California, Berkeley, CA) and grown as described [23]. For the *Lm* experiment, uninfected cells (referred to as 0-hr time point) were collected after culturing without *Lm* for 2 hrs; in contrast, the unstimulated cells in the LPS experiment were collected immediately after isolation.

Mouse macrophages were prepared from the bone marrow of 6-week-old C57BL/6, IRF3^-/-^, or IFNAR^-/-^ mice as described [24,25], and were stimulated with lipid A (100 ng/mL) (Sigma) after 6 days of differentiation.

### RNA isolation, library preparation, and sequencing

Human monocyte RNA was purified using the RNeasy Mini Kit (Qiagen) according to the manufacturer’s protocol. Strand-specific libraries were prepared using 120 ng RNA input according to the “deoxyuridine triphosphate (dUTP)” method [26]. Mouse macrophage experiments involved analyses of chromatin-associated RNAs, as previously described [25]. A HiSeq 2000 (Illumina) was used for sequencing, with a single end sequencing length of 50 nucleotides. Sequencing data have been submitted to GEO under accession number GSE60216.

### Bioinformatic analyses

All bioinformatic analyses were conducted using the Galaxy platform [27]. Reads were aligned to the human GRCh37 or mouse mm9 reference genomes with Tophat [28] using most default parameters. Alignments were restricted to uniquely mapping reads with two possible mismatches permitted. RPKM (reads per kilobase pair per million mapped reads) were calculated using Seqmonk (http://www.bioinformatics.babraham.ac.uk/projects/seqmonk/). Coexpressed gene classes were evaluated with Cluster3 by applying k-means clustering to mean-centered log2(RPKM) expression values [29]. Statistically significant gene expression differences were evaluated using DESeq [30]. Mouse orthologs of human genes were identified using BLAST (http://blast.ncbi.nlm.nih.gov/Blast.cgi). Pscan was used to detect DNA motifs overrepresented in each class between nucleotides -450 and +50 relative to the transcription start site [31].

## Results

### Gene expression cascades induced in monocytes by LPS and *Lm*

An attractive starting point toward a full understanding of age-related differences in immune responses is to employ RNA-seq to carefully examine mRNA transcript levels following stimulation or infection of defined cell types. Toward this goal, peripheral blood monocytes were obtained from healthy young adults and healthy older adults. In addition, neonatal monocytes were obtained from umbilical cord blood samples; cord blood is known to consist almost exclusively of neonatal cells [32]. Monocytes from three individuals of each age group were stimulated with LPS or infected with *Lm*. For the LPS experiments, samples were collected 0, 1, and 6 hrs post-stimulation. For the *Lm* experiments, samples were collected 0, 2, and 6 hrs post-infection. After mRNA isolation and cDNA library preparation, RNA-seq was performed. The number of mapped reads ranged from 3.4 x 10^6^ to 1.3 x 10^7^ per sample.

**Fig 1:**
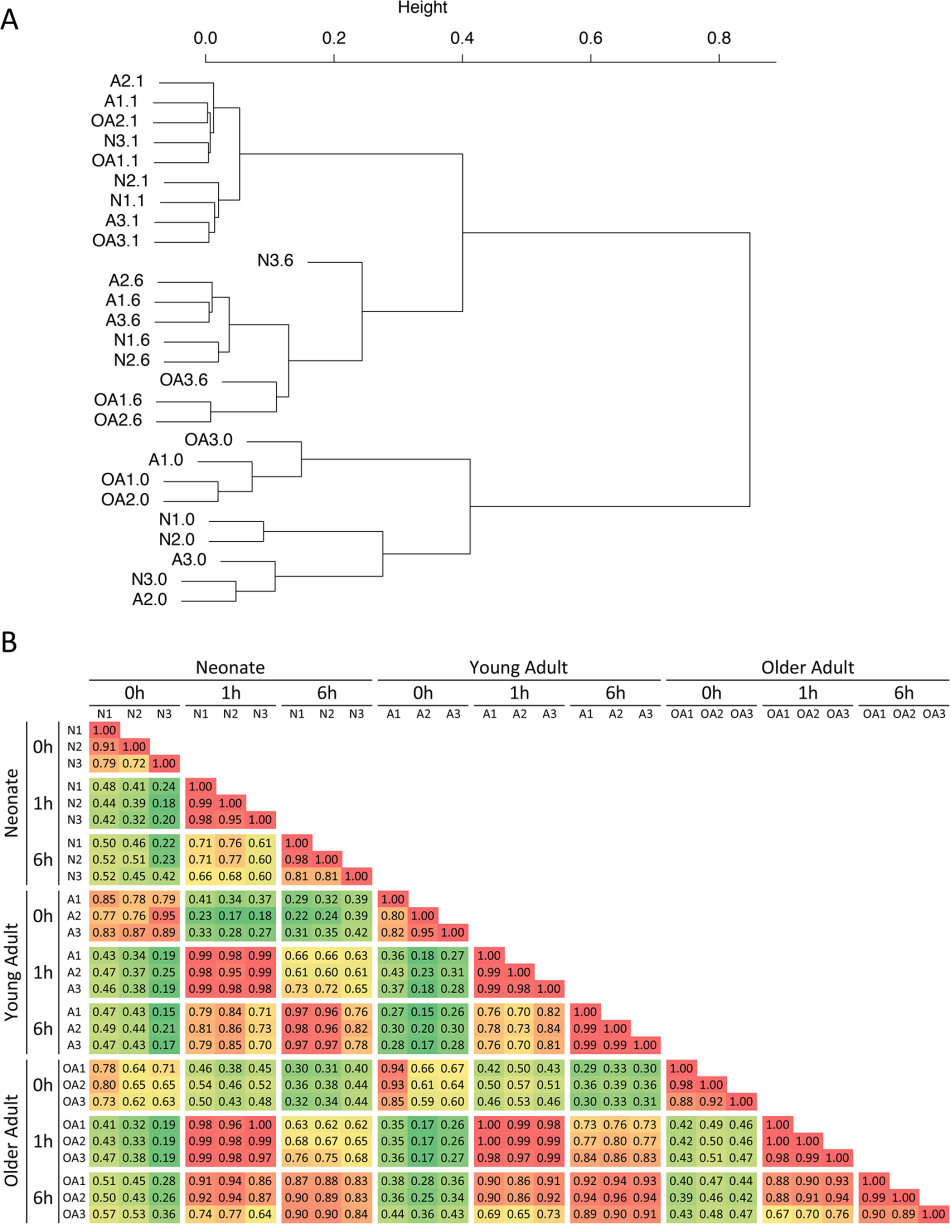
Hierarchical clustering of LPS-stimulated monocyte transcriptomes from human neonates, adults, and older adults. (A) RNA-seq experiments were performed with three independent human monocyte samples from cord blood (N), young adult peripheral blood (A), and older adult peripheral blood (OA) stimulated with LPS for 0, 1, and 6 hrs. Hierarchical clustering was performed with the 1147 genes found to be induced by at least 5-fold at the 1- or 6-hr time point in at least one sample and with an induced RPKM of at least 4 (genes smaller than 200 bp were also excluded from the analysis). Sample codes correspond to the age abbreviation followed by the sample number (1 through 3 for each age); the time point (0, 1, or 6 hr) is indicated after the period. Inducible transcriptomes exhibit strong time-dependent clustering, with much less age-dependent clustering. (B) Pearson correlation values (R) used for the hierarchical clustering in panel A are shown. Each time point from each sample was compared to every other sample and time point. R values are color-coded from low (green) to high (red). Samples on the X and Y axes are grouped first according to age group, then time point (0, 1, or 6), and then sample number (1–3).

An examination of the data sets from the LPS experiment identified 1147 annotated RefSeq genes that were induced by at least five-fold at the 1- or 6-hr time point (relative to the unstimulated sample) in at least one sample from any age group, and that exhibited a transcript level exceeding four RPKM following induction. To examine the relationship between the different time points and age groups in the response to LPS, hierarchical clustering was performed with these 1147 genes (Fig 1A). This analysis revealed that each of the nine samples from a given time point was more closely related to the other samples from the same time point than to any sample from the other two time-points. The most significant difference that showed a possible relationship to age was that the three unstimulated samples from older adults (OA1.0, OA2.0, and OA3.0) and one young adult unstimulated sample (A1.0) clustered separately from the remaining unstimulated samples from young adults and neonates.

Small age-related differences were also observed with the 6-hr time-point data, in that, with only one exception (neonatal sample N3.6), each age group clustered separately from the others. In contrast, the nine 1-hr time-point samples correlated closely, with no apparent age-related differences. The Pearson correlation values (R values) used for the hierarchical clustering are shown in Fig 1B. These results provide initial evidence that the vast majority of LPS-induced genes are induced similarly in the three age groups.

**Fig 2:**
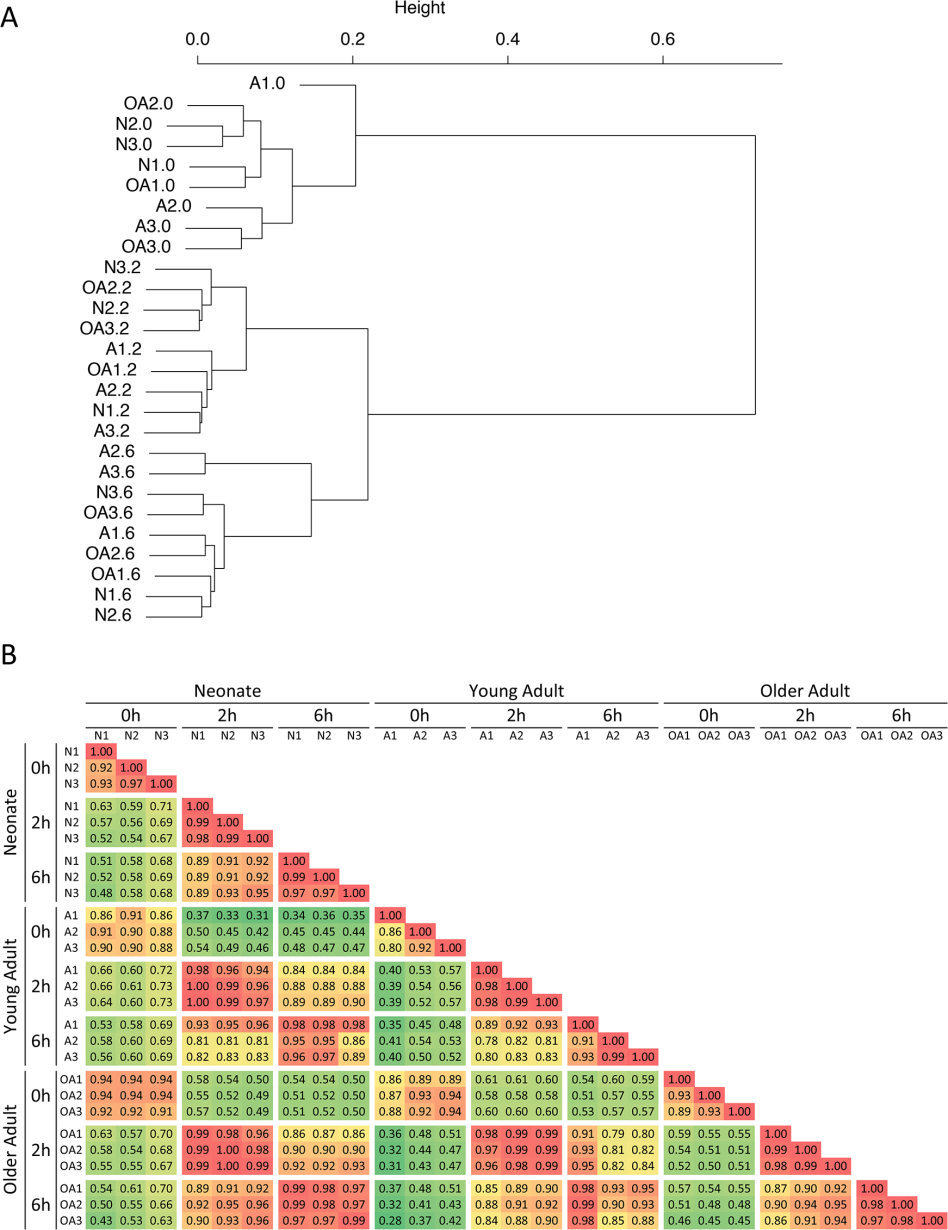
Hierarchical clustering of *Lm*-infected monocyte transcriptomes from human neonates, adults, and older adults. (A) RNA-seq experiments were performed with three independent human monocyte samples from cord blood (N), young adult peripheral blood (A), and older adult peripheral blood (OA) infected with *Lm* for 0, 2, and 6 hrs. Hierarchical clustering was performed with the 865 genes found to be induced by at least 5-fold at the 2- or 6-hr time point in at least one sample and with an induced RPKM of at least 4 (genes smaller than 200 bp were also excluded from the analysis). Sample codes correspond to the age abbreviation followed by the sample number (1 through 3 for each age); the time point (0, 2, or 6 hr) is indicated after the period. Inducible transcriptomes exhibit strong time-dependent clustering, with much less age-dependent clustering. (B) Pearson correlation values (R) used for the hierarchical clustering in panel A are shown. Each time point from each sample was compared to every other sample and time point. R values are color-coded from low (green) to high (red). Samples on the X and Y axes are grouped first according to age group, then time point (0, 2, or 6), and then sample number (1–3).

Examination of the *Lm* data sets identified 865 annotated RefSeq genes that were induced by at least five-fold at the 2-hr or 6-hr time point in at least one sample, and that exhibited a transcript level exceeding four RPKM following induction. The hierarchical clustering results and the Pearson correlation values revealed even stronger correlations between age groups at each time point than were observed with the LPS data (Fig 2). That is, although strong time-dependent clustering was observed, no consistent age-related differences were observed at any of the time points.

### K-means cluster analysis of LPS- and *Lm*-induced genes

**Fig 3:**
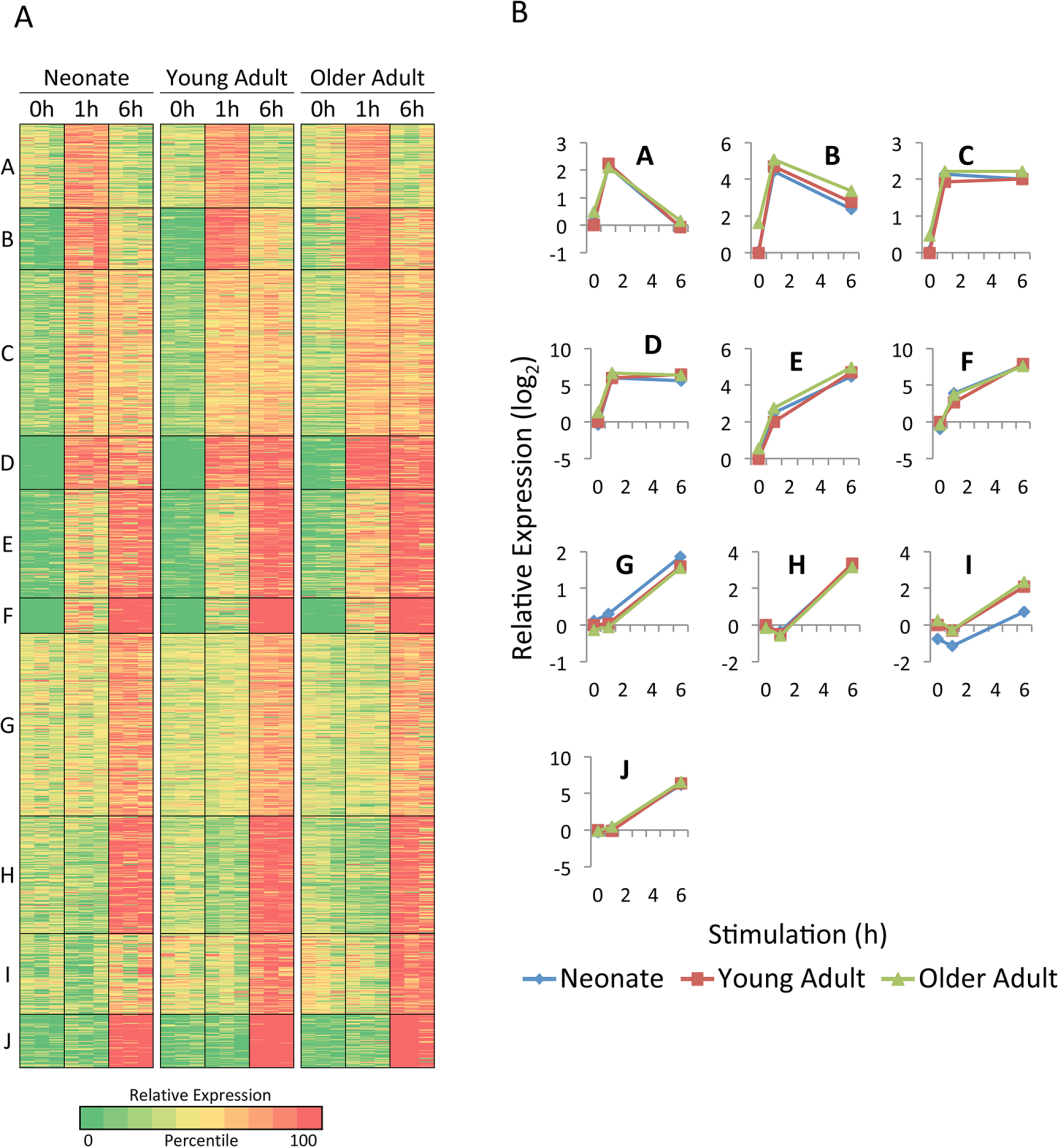
Analysis of LPS-induced genes in monocytes by K-means cluster analysis. (A) The 1147 genes that exceeded 200 bp in length, exhibited an RPKM of at least 4 in one sample, and were induced by LPS by at least 5-fold in the same sample were divided into 10 clusters by k-means cluster analysis, which considers similarities in transcript levels for each gene across all 27 samples (3 age groups, 3 samples for each age group, and 3 time points for each sample). The three independent samples are shown in parallel for each age group. Colors indicate the percentile of the relative expression level (based on the log-transformed mean-centered RPKM for each gene), as indicated at the bottom. (B) The average relative transcript levels for genes within each cluster are shown for each age group (neonates, blue diamonds; young adults, red squares; older adults, green triangles).

To extend the analysis of age-related differences in inducible gene expression, k-means clustering was used to define groups of genes that exhibited similar expression patterns among the three age groups and three time points. The k-means algorithm considers induction kinetics, induction magnitudes, and differences among age groups. Fig 3A shows the results obtained when the 1147 LPS-induced genes (using the average expression values from the three independent samples analyzed for each age group and each time point) were assigned to one of ten distinct clusters. As expected on the basis of the hierarchical clustering, extensive similarities were apparent in the three age groups in almost all of the clusters. The similarities are also apparent in line graphs showing the average relative expression levels for all genes in a given cluster (Fig 3B).

Only one cluster (Cluster I) was identified that showed substantial age-related differences (Fig 3). Genes in this cluster were generally expressed at a lower level in both unstimulated and LPS-stimulated monocytes from neonates in comparison to the young adult and older adult samples. Although the average induction magnitude for genes in this cluster was comparable among the age groups, the average expression level of these genes was significantly lower in neonates than in young adults at all three time points.

**Fig 4:**
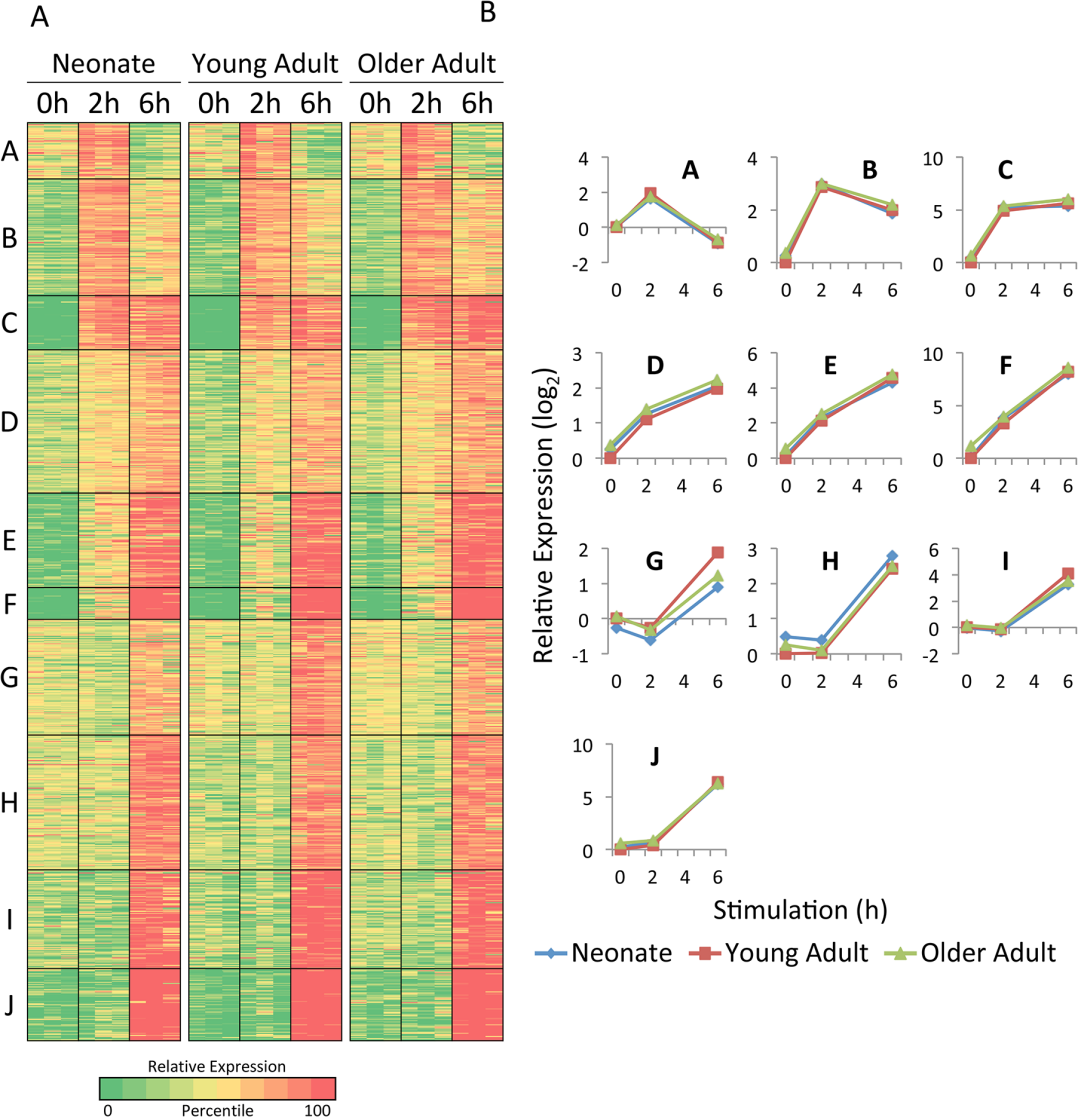
Analysis of *Lm*-induced genes in monocytes by K-means cluster analysis. (A) The 865 genes that exceeded 200 bp in length, exhibited an RPKM of at least 4 in one sample, and were induced by *Lm* infection by at least 5-fold in the same sample were divided into 10 clusters by k-means cluster analysis, which considers similarities in transcript levels for each gene across all 27 samples (3 age groups, 3 samples for each age group, and 3 time points for each sample). The three independent samples are shown in parallel for each age group. Colors indicate the percentile of the relative expression level (based on the log-transformed mean-centered RPKM for each gene), as indicated at the bottom. (B) The average relative transcript levels for genes within each cluster and are shown for each age group (neonates, blue diamonds; young adults, red squares; older adults, green triangles).

K-means clustering of the *Lm*-induced genes also revealed extensive similarities among the three age groups (Fig 4). Only one cluster (Cluster G) showed slightly reduced average expression in the neonatal and older adult samples in comparison to the young adult samples.

### Analysis of genes exhibiting statistically significant expression differences

Because the clustering results described above revealed extensive similarities with limited age-related differences, we envisioned that meaningful insights would require the use of defined parameters to identify genes that exhibited the greatest differential expression. Toward this end, we first focused our attention on genes induced to a statistically significant extent (p<0.01) that also exhibited differential expression between neonates and young adults at a high level of statistical significance (p<0.01). Only 118 of the 1147 LPS-induced genes met these criteria.

**Fig 5:**
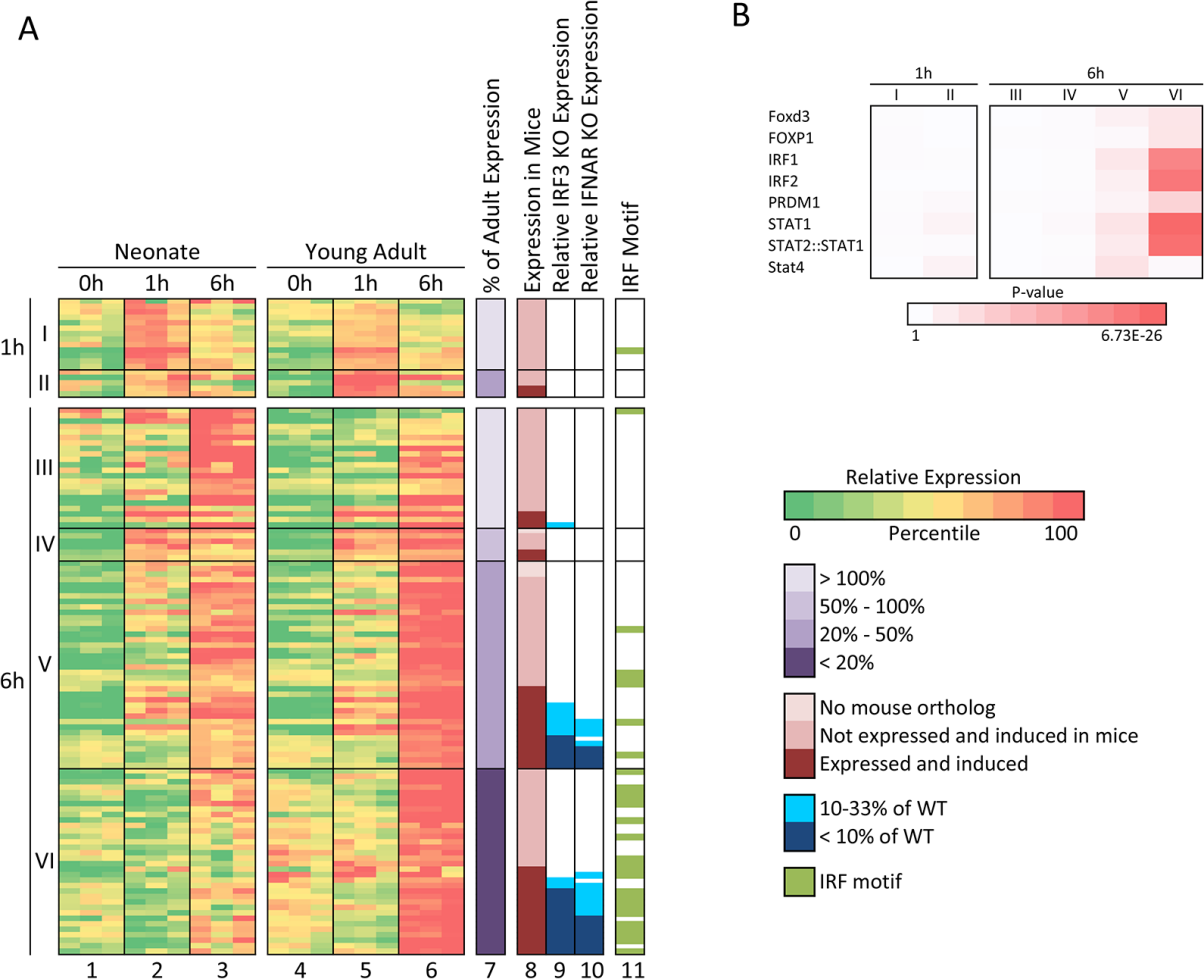
Genes that exhibit the greatest expression deficit in LPS-stimulated cord blood monocytes in comparison to adult monocytes are regulated by IRF3 and/or Type I IFNs. (A) LPS-induced genes exhibiting statistically significant differential expression in neonates and adults (n = 118) were grouped according to the time point at which their maximum transcript levels were observed (1 or 6 hrs). They were then grouped according to their relative maximum transcript levels in cord blood (neonates) versus young adults. Induced genes with a higher maximum transcript level in neonates than young adults (with statistically significant differential expression) are included in classes I (1-hr peak) and III (6-hr peak) (column 7). Genes exhibiting a maximum transcript level in neonates that was 50–100% of the young adult transcript level (but with statistically significant differential expression) are included in class IV (no genes with peak transcript levels at 1-hr fit this criterion). Genes exhibiting a maximum transcript level in neonates that was 20–50% of the young adult transcript level are in classes II (1-hr) and V (6-hr). Genes with a maximum transcript level in neonates below 20% of the young adult transcript level are in class VI. The differential expression of six of these genes was confirmed by quantitative RT-PCR (data not shown). Columns 1–6 show the relative transcript levels (based on the log-transformed mean-centered RPKM) for these 118 classified genes in all samples and all time points from both neonates and young adults. Column 8 indicates genes that lack obvious mouse orthologs (lightest pink), genes that contain mouse orthologs that are either not expressed or not induced in mouse bone marrow-derived macrophages (dark pink), and genes containing mouse orthologs that are both expressed and induced by LPS (red). Columns 9 and 10 show relative expression of the mouse ortholog of the human gene in Lipid A-stimulated macrophages from IRF3^-/-^ and IFNAR^-/-^ mice, respectively (see blue scale at right). Note that these columns are only relevant for genes shown in red in Column 8. Column 11 indicates genes with promoters that contain an IRF1 transcription factor binding motif between -450 and +50 bps relative to the transcription start site. (B) Enrichment of transcription factor binding sites determined using the Pscan program is shown for each gene class from panel A. Color intensity is proportional to the negative log(p-value).

The 118 genes (gene identities listed in S1 Fig) were separated into groups according to the time point at which their maximum mRNA level was observed (Fig 5A: 1-hr peak expression for Groups I and II; 6-hr peak expression for Groups III-VI). The genes were then further grouped according to their expression level in neonates relative to their expression level in young adults (Fig 5A, column 7). (For this calculation, the baseline and maximum expression levels in young adults were defined as 0% and 100%, respectively; the maximum expression level in neonates was then determined as a percentage relative to that range.) This analysis revealed 35 genes that exhibited enhanced expression in the neonatal samples (Groups I and III, lightest shade of purple) and 83 genes that exhibited reduced expression (Groups II, IV, V, and VI, three darker shades of purple). Group VI contains the 34 genes that exhibited the greatest difference between neonates and young adults. For these genes, the maximum LPS-induced mRNA level in neonates was less than 20% of the maximum level observed in young adults.

**Fig 6:**
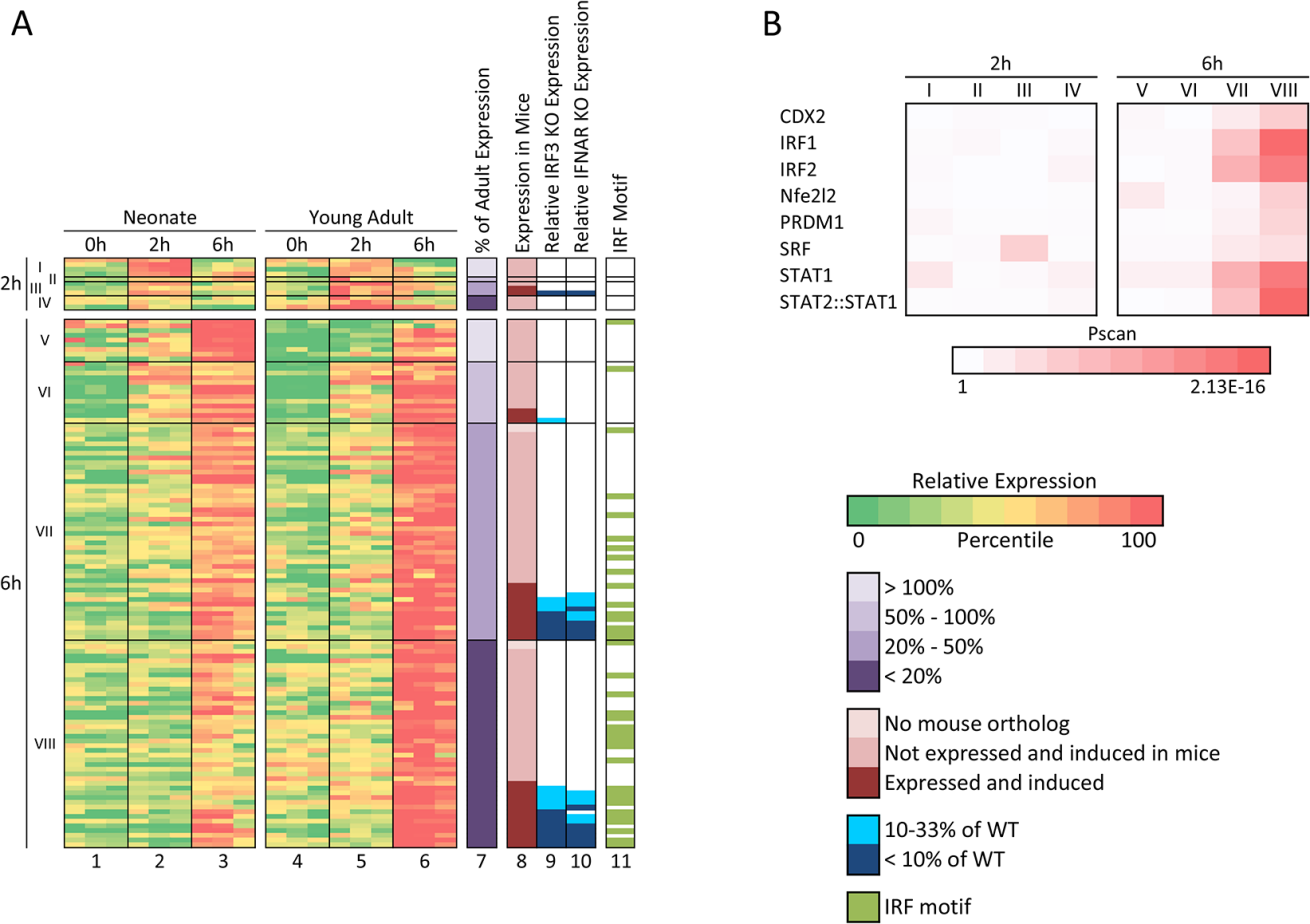
Genes that exhibit the greatest expression deficit in *Lm*-stimulated cord blood monocytes in comparison to adult monocytes are regulated by IRF3 and/or Type I IFNs. (A) *Lm*-induced genes exhibiting statistically significant differential expression in neonates and young adults (n = 123) were grouped according to the time point at which their maximum transcript levels were observed (2 or 6 hrs). They were then grouped according to their relative maximum transcript levels in cord blood (neonates) versus young adults. Induced genes with a higher maximum transcript level in neonates than young adults (with statistically significant differential expression) are included in classes I (2-hr peak) and V (6-hr peak) (column 7). Genes exhibiting a maximum transcript level in neonates that was 50–100% of the young adult transcript level (but with statistically significant differential expression) are included in classes II (2-hr) and VI (6-hr). Genes exhibiting a maximum transcript level in neonates that was 20–50% of the young adult transcript level are in classes III (2-hr) and VII (6-hr). Genes with a maximum transcript level in neonates below 20% of the young adult transcript level are in classes IV (2-hr) and VIII (6-hr). Columns 1–6 show the relative transcript levels (based on the log-transformed mean-centered RPKM) for these 123 classified genes in all samples and all time points from both neonates and young adults. Column 8 indicates genes that lack obvious mouse orthologs (lightest pink), genes that contain mouse orthologs that are either not expressed or not induced in mouse bone marrow-derived macrophages (dark pink), and genes containing mouse orthologs that are both expressed and induced by LPS (red). Columns 9 and 10 show relative expression of the mouse ortholog of the human gene in Lipid A-stimulated macrophages from IRF3^-/-^ and IFNAR^-/-^ mice, respectively (see blue scale at right). Note that these columns are only relevant for genes shown in red in Column 8. Column 11 indicates genes with promoters that contain an IRF1 transcription factor binding motif between -450 and +50 bps relative to the transcription start site. (B) Enrichment of transcription factor binding sites determined using the Pscan program is shown for each gene class from panel A. Color intensity is proportional to the negative log(p-value).

A parallel analysis with the *Lm* samples identified 123 genes (listed in S2 Fig) that were inducible and differentially expressed between neonates and young adults with a high level of statistical significance (p<0.01 for both induction and differential expression). Grouping of these genes using the same strategy as above revealed 13 genes that were expressed more highly in neonates than young adults (Fig 6A, Groups I and V) and 110 genes that were expressed more highly in young adults than neonates (Groups II-IV and VI-VIII). Forty-seven of these later genes exhibited mRNA levels in neonates that were less than 20% of the young adult levels (Groups IV and VIII).

### A prominent role for IRF3 and Type I IFN signaling in the neonate-adult differences

To gain insight into the mechanisms responsible for differential gene expression in neonatal and young adult monocytes, we first examined the requirements for expression of the mouse orthologs of the differentially expressed genes. This analysis took advantage of a large number of RNA-seq data sets that have been generated in our laboratory using mouse bone marrow-derived macrophages stimulated with the Lipid A component of LPS. This collection of data sets includes kinetic analyses of lipid A-induced gene expression in macrophages from a variety of mutant mouse strains lacking key signaling molecules or transcription factors thought to be important for inducible transcription ([25] and unpublished results).

By examining the expression requirements for the mouse orthologs of the genes that were differentially expressed in human neonates and young adults, evidence was obtained that these genes frequently require the transcription factor IRF3 or Type I IFN receptor signaling. That is, many of the age-dependent differentially expressed genes were expressed at substantially reduced levels in *Irf3* ^*-/-*^ and/or *Ifnar* ^*-/-*^ macrophages stimulated with Lipid A.

To document the extent to which IRF3 and IFNAR signaling might contribute to the differential expression of LPS-induced genes in neonates and adults, human genes for which mouse orthologs could clearly be identified (114 of 118 genes; Fig 5A, column 8, dark pink and red) were first separated from the small number of genes lacking obvious mouse orthologs (Fig 5A, column 8, lightest pink). Then, the RNA-seq data sets were analyzed to identify genes that were both expressed (RPKM > 4 when maximally expressed) and induced (>5-fold) in both the human monocytes and wild-type mouse macrophages. The 38 genes that met these criteria (Fig 5A, column 8, red) were then evaluated for their dependence on IRF3 and IFNAR in mouse bone marrow-derived macrophages stimulated with Lipid A. The results revealed IRF3-dependence for 14 of the 16 genes in Group VI (Fig 5A, column 9, dark blue if <10% of the wild-type expression level in *Irf3* ^*-/-*^ macrophages and light blue if 10–33% of the wild-type level in *Irf3* ^*-/-*^ macrophages). 14 of the 16 genes also exhibited reduced expression in *Ifnar* ^*—/-*^ macrophages (column 10). IRF3- and/or IFNAR-dependence was also observed for most Group V genes for which mouse orthologs were both expressed and induced in mouse macrophages (Fig 5A).

As an independent strategy, a transcription factor binding site motif analysis was performed using the Pscan program [31] with the promoter regions of all genes in Groups I through VI. The goal of this analysis was to identify transcription factors whose binding sites are over-represented in the promoters of specific clusters of genes. The small number of transcription factors for which significant enrichment was observed are shown in Fig 5B. Transcription factor binding motif enrichment generally was not observed for Groups I through V. However, highly significant enrichment of binding sites for IRF1, IRF2, STAT1, and a STAT2:STAT1 heterodimer was found at the promoters of Group VI genes (Fig 5B). The IRF1 and IRF2 binding sites used by the Pscan program are similar to the experimentally defined consensus IRF3 binding motif [33], which is not assessed by Pscan. Importantly, IRF and STAT motifs were identified in the promoters of the vast majority of Group VI genes, including most genes whose mouse orthologs could not be examined for IRF3 and IFNAR dependence due to lack of inducible expression in both mice and humans (Fig 5A, column 11).

Thus, both the functional analysis and motif analysis strongly support the hypothesis that reduced activation of IRF3- and IFNAR-dependent genes explains most gene expression differences between neonatal and adult monocytes. It is noteworthy that a previous study which documented reduced IRF3 activity in neonatal dendritic cells found that neonatal and adult cells were similarly responsive to IFNβ stimulation, suggesting that the reduced expression of IFNAR-dependent genes is due to reduced IRF3 activity (resulting in reduced IFNβ expression) rather than a reduction in IFNAR signaling [15].

Consistent with the analysis of the LPS-induced genes, mouse orthologs of the human genes that exhibited differential expression upon *Lm* infection were generally found to exhibit IRF3- and/or IFNAR-dependence (Fig 6A). Furthermore, binding sites for IRF1, IRF2, STAT1, and the STAT2:STAT1 heterodimer were greatly enriched in the Group VIII genes and to a lesser extent in Group VII genes (Fig 6A and 6B). Thus, although IRF3 is thought to be activated by different pathways in LPS-stimulated and *Lm*-infected cells [34,35], a common reduction in IRF3 activity is likely to be responsible for the strongest gene expression differences between neonatal and adult monocytes.

### Low-level inflammation in older adults

**Fig 7:**
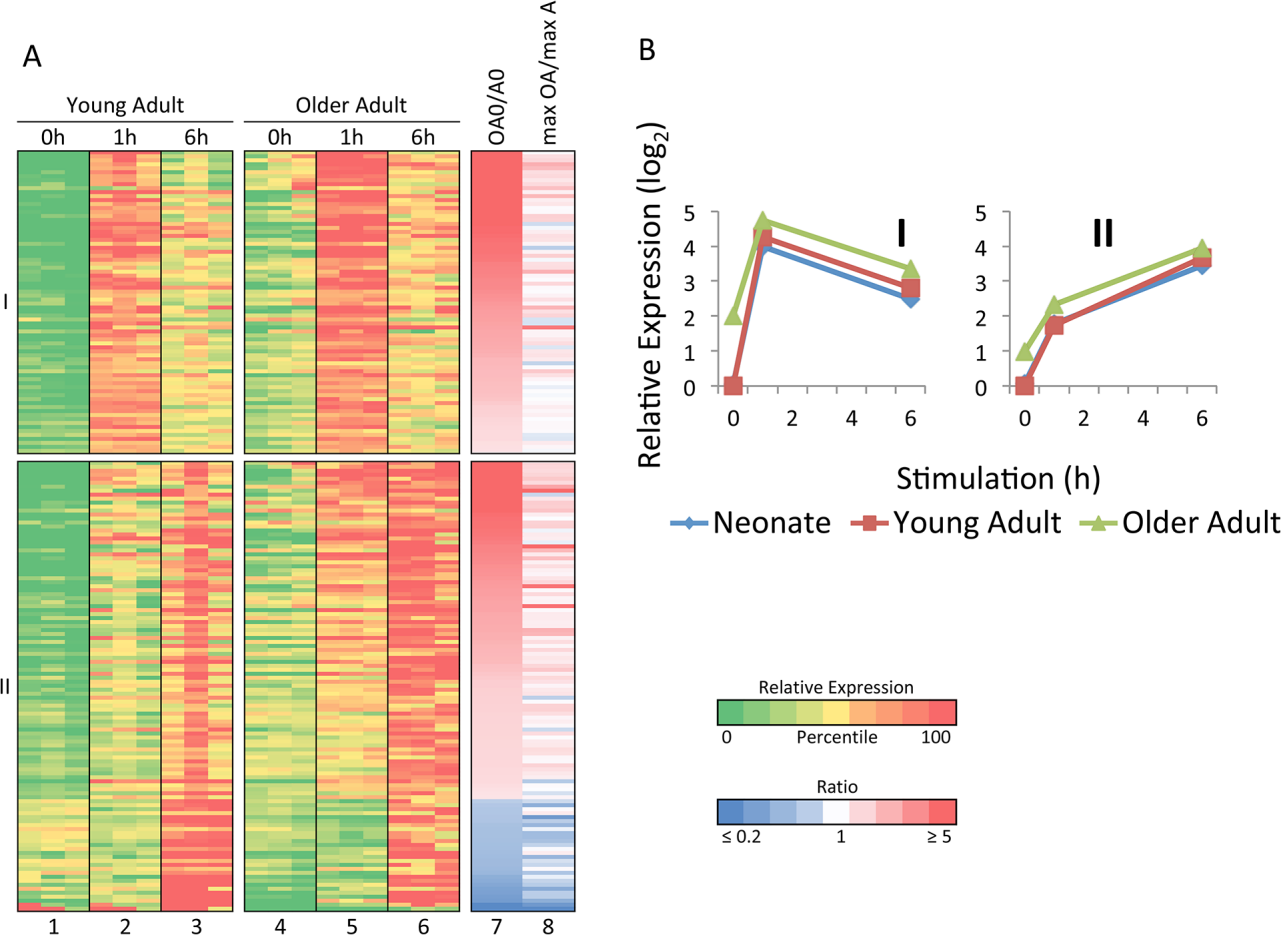
Elevated expression of a broad range of inflammatory genes prior to stimulation of freshly isolated monocytes from older adults. (A) LPS-induced genes exhibiting differential basal expression between adults and older adults (n = 189) are grouped according to maximum mRNA level. Columns 7 and 8 show the ratio of transcript levels between older adults and young adults before stimulation and at maximum transcript levels, respectively. (B) The average relative transcript levels within each cluster and for each age are shown.

To evaluate gene expression differences between young adults and older adults, we first used the strategy described above to identify differentially induced genes. This analysis revealed smaller differences in transcriptional induction than were observed when comparing the neonatal and young adult profiles (data not shown), suggesting that the pathways involved in the responses to LPS and *Lm* in monocytes in young adults and older adults are highly similar. Instead, the largest differences were observed when examining transcript levels for inducible genes prior to stimulation. Specifically, 189 LPS-induced genes (>5-fold induction magnitude; induction significance p<0.01; maximum induced transcript level >4 RPKM) exhibited transcript levels that were significantly different (p<0.01) in unstimulated cells from young adults in comparison to older adults (Fig 7A; gene list in S3 Fig). For these 189 genes, Fig 7A, column 7 shows the ratio of the unstimulated transcript level in older adults to that in younger adults (OA0/A0). In this figure, the genes are grouped on the basis of their time point of maximum expression, and the genes were then rank-ordered by the ratio of the unstimulated transcript level. This analysis revealed that a large majority of the differentially expressed genes are expressed at an elevated level in unstimulated cells from older adults (shown as shades of red, see vertical color scale at right). In fact, 52% of the differentially expressed genes exhibited unstimulated transcript levels in older adults that were at least 3-fold higher than in young adults, whereas only 3% exhibited transcript levels that were at least 3-fold higher in young adults than in older adults. Similar results were observed in the *Lm* experiment (data not shown), but the number of genes showing differential expression was lower, probably because the unstimulated cells for the *Lm* experiment were cultured for 2 hrs prior to collection, whereas the unstimulated cells in the LPS experiment were collected without culturing.

Importantly, although relatively large differences in expression between young adults and older adults were observed in the unstimulated cells, the magnitudes of the differences were generally lower after stimulation. This is apparent in Fig 7A, column 8 (max OA/max A), which shows the ratio between the maximum induced transcript levels in older adults versus young adults. Because the same color scale is used for columns 7 and 8, it is readily apparent that the transcript level ratios move toward 1 after stimulation for many genes that are differentially expressed prior to stimulation. Fig 7B, which displays average transcript levels for all genes in Groups I and II, also shows that transcript levels in older adults were elevated to a greater extent prior to stimulation than after stimulation. It is important to note, however, that although the differences between young adults and older adults are smaller after stimulation, many of these inflammatory genes are still expressed slightly higher after stimulation in the older adult samples than in the young adult samples. This differential expression causes the older adult 6-hr time point samples to cluster separately from the young adult and neonatal 6-hr samples in Fig 1A, although the separation is smaller that than observed with the unstimulated samples (see Fig 1A). Thus, an inflammatory state is readily apparent in unstimulated monocytes from older adults. This inflammatory state in unstimulated cells may influence transcript levels observed after stimulation or infection, but to a limited extent relative to the differences observed in the basal state.

## Discussion

The diminished capacity of human neonates and older adults to mount an immune response to infectious agents has been well documented [1,2]. However, because of the complexity of the human immune system and limitations in the experimental approaches that are available for studying immune responses in humans, insights into the underlying mechanisms have been difficult to obtain. One starting point toward a mechanistic understanding can be characterized as reductionist, in which the goal is to first delineate age-related differences intrinsic to defined immune cell types in an *ex vivo* setting, with subsequent experiments focusing on how these intrinsic differences contribute to clinical observations in the far more complex *in vivo* setting.

In this study, RNA-seq was used to examine the intrinsic response of blood monocytes to LPS stimulation and *Lm* infection. The improved dynamic range of the RNA-seq method in comparison to microarray methods [21] led to the expectation that the results might reveal extensive differences among the age groups. Given this expectation, the most striking finding is perhaps the extensive similarity in both constitutive and inducible gene expression. The results suggest that a single mechanism–variable induction of IRF3 –may be responsible for most and perhaps all differences between neonatal and young adult monocytes. Another defined mechanism, variable low-level inflammation prior to induction, may explain most of the differences between young adults and older adults.

Our results strengthen previous evidence that reduced IRF3 activity makes a major contribution to the deficient innate responses of neonates to infectious stimuli [15]. The previous study was performed with LPS-stimulated dendritic cells differentiated from cord blood or adult peripheral blood, whereas the current study was performed with freshly isolated monocytes stimulated with LPS or infected with *Lm*. In the previous study, a large number of IRF3- and Type 1 IFN-dependent genes were found to be expressed at reduced levels in neonates. The reduced expression of these genes was attributed to reduced IRF3 activity because the neonatal and adult cells responded similarly to direct stimulation with IFNβ. Reduced IRF3 activity would lead to a broad reduction in the expression of IFN-dependent genes because IRF3 is critical for the initial induction of *IFNB* transcription in LPS-stimulated cells.

Interestingly, the previous study found that IRF3 translocated to the nucleus similarly in neonatal and adult cells, and its *in vitro* DNA-binding activity was similarly induced [15]. However, its ability to bind endogenous target genes was reduced, suggesting that an additional event–possibly an additional post-translational modification–is needed for binding to target genes and may be reduced in neonatal cells. Of relevance, a separate study identified a major defect in IRF7 activation in neonatal plasmacytoid dendritic cells and, in this cell type, a defect in nuclear translocation of IRF7 was observed in neonates [36]. An additional clue into the underlying mechanism is our finding of a similar deficiency in both LPS-stimulated and *Lm*-infected cells. LPS and *Lm* activate IRF3 via different signaling pathways–the TRIF pathway for LPS and the STING pathway for *Lm* [34,35]–suggesting that the reduced IRF3 activity in neonatal cells involves a mechanism that influences the activation of IRF3-dependent genes via both of these pathways.

In addition to elucidating the specific mechanism, it will be important to understand why this difference exists between neonatal and adult monocytes. The simplest model is that neonatal monocytes are fundamentally different from adult monocytes and represent a developmentally distinct monocyte subtype. However, this model predicts that prominent gene expression differences would be observed prior to stimulation. The differentially expressed genes would be expected to include cell-surface markers that define different myeloid cell populations and genes that might help regulate IFN responses. Surprisingly, the expression profiles of the unstimulated monocytes from neonates and adults were remarkably similar (data not shown), with no large differences suggesting that they represent different myeloid subtypes, and no differences that would be predictive of the differential induction of IRF3-dependent genes.

One possible explanation for this apparent paradox is that the differences between neonatal and adult monocytes are due to the differential expression of micro-RNAs or long noncoding RNAs, which were not examined in this analysis. However, the differential expression or processing of non-coding RNAs would be expected to require the differential expression of transcription factors that regulate the non-coding RNA genes, or the differential expression of processing enzymes; these protein-coding genes would have been included in our analysis. Differences in alternative pre-mRNA splicing also were not examined in our analysis. Once again, differential splicing would be expected to require the differential expression of genes encoding splicing factors. A more likely possibility is that the pronounced difference in the induction of IRF3-dependent genes is regulated by genes whose expression levels vary by only a small and statistically insignificant amount.

Because the RNA-seq profiles failed to provide evidence that the neonatal and adult cells represent developmentally distinct monocyte subtypes, the neonatal-adult differences may instead be due to environmental differences that act on the fully differentiated cells to influence their capacity to induce IRF3 activity. Such a mechanism would need to influence IRF3’s capacity for induction for a prolonged time period, because the IRF3 difference has been observed in dendritic cells differentiated for several days *in vitro* [15]. This environmental difference may lead to small and stable differences in the expression of genes that regulate IRF3 activity. Alternatively, the neonatal microenvironment may alter the structure of chromatin at IRF3-dependent genes, resulting in a reduced capacity for IRF3 binding in response to a stimulus.

To summarize, the results of this study will help guide future efforts to understand the mechanisms responsible for the immune deficiencies observed in neonates and older adults. The results suggest that the intrinsic properties of blood monocytes are remarkably stable throughout life and vary to only a limited extent. The reduced capacity of neonatal monocytes to activate IRF3-dependent genes could play an important role in the deficient response of neonates to many microbial pathogens. Furthermore, the low-level inflammation that is readily apparent in monocytes from older adults could also influence anti-microbial responses. RNA-seq studies to quantitatively characterize intrinsic age-related differences in other innate and adaptive immune cell types should provide additional insights and should ultimately suggest strategies to enhance immune responses in deficient populations.

## Supporting Information

S1 Fig LPS-induced genes exhibiting statistically significant differences in transcript levels in cord blood and young adult monocytes. An expanded version of Fig 5A is shown, which includes the identities of the LPS-induced genes that are differentially expressed in cord blood and young adult monocytes. RefSeq IDs and gene names are shown for human genes and their mouse orthologs.
(TIF)

S2 Fig *Lm*-induced genes exhibiting statistically significant differences in transcript levels in cord blood and young adult monocytes. An expanded version of Fig 6A is shown, which includes the identities of the *Lm*-induced genes that are differentially expressed in cord blood and young adult monocytes. RefSeq IDs and gene names are shown for human genes and their mouse orthologs.
(TIF)

S3 Fig LPS-induced genes that exhibit statistically significant differences in basal transcript levels in monocytes from young and older adults. An expanded version of Fig 7A is shown, which includes the identities of LPS-induced genes that are differentially expressed in unstimulated young and older adult monocytes. Human RefSeq IDs and gene names are shown.
(TIF)
