# Supplementary figures and images for: Age-Related Gene Expression Differences in Monocytes from Human Neonates, Young Adults, and Older Adults
Source: PLoS One. 2015 Jul 6;10(7):e0132061. doi: 10.1371/journal.pone.0132061 (PMC4493075; doi:10.1371/journal.pone.0132061)

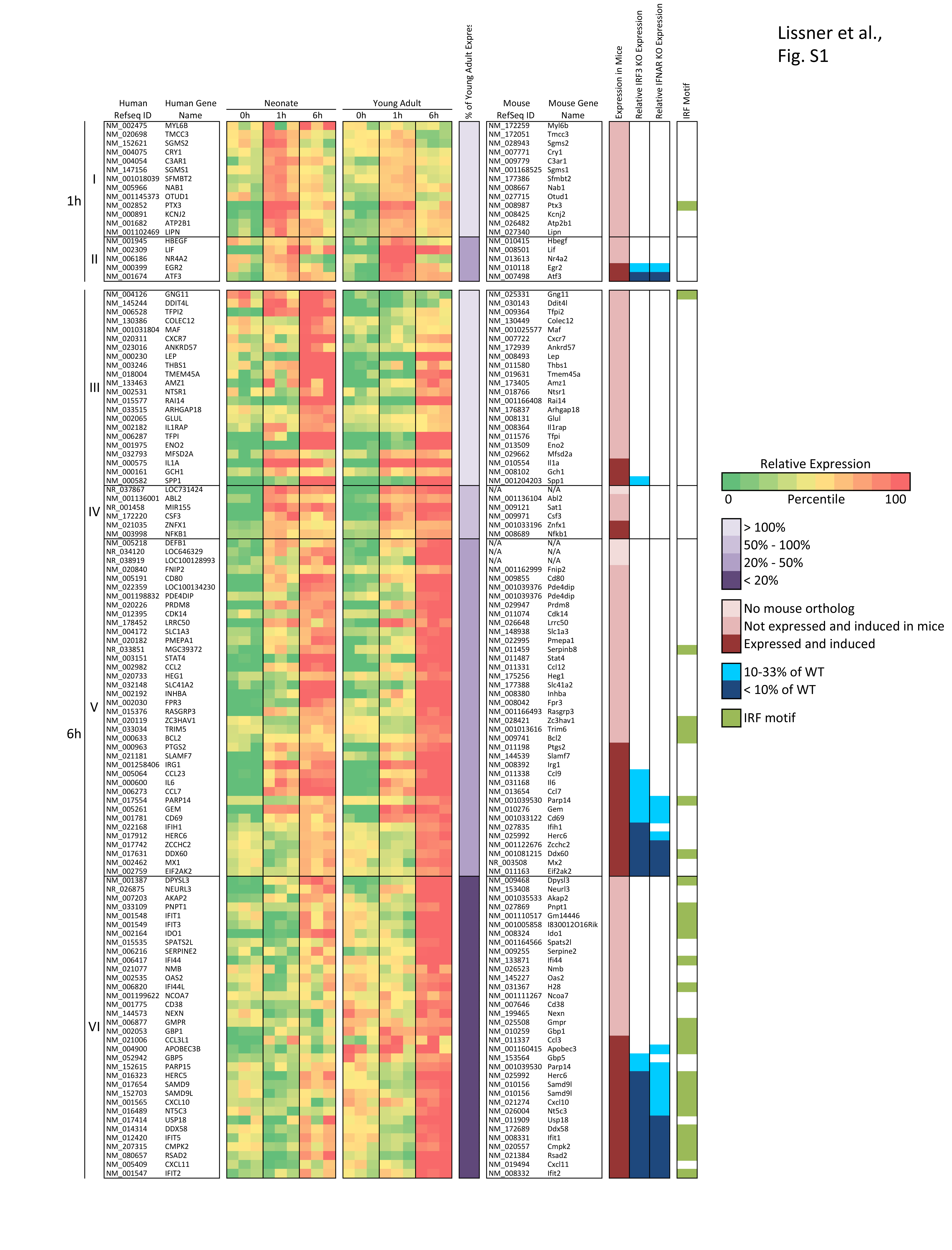

Supplement: S1 Fig — An expanded version of Fig 5A is shown, which includes the identities of the LPS-induced genes that are differentially expressed in cord blood and young adult monocytes. RefSeq IDs and gene names are shown for human genes and their mouse orthologs. (TIF) [file pone.0132061.s001.tif]

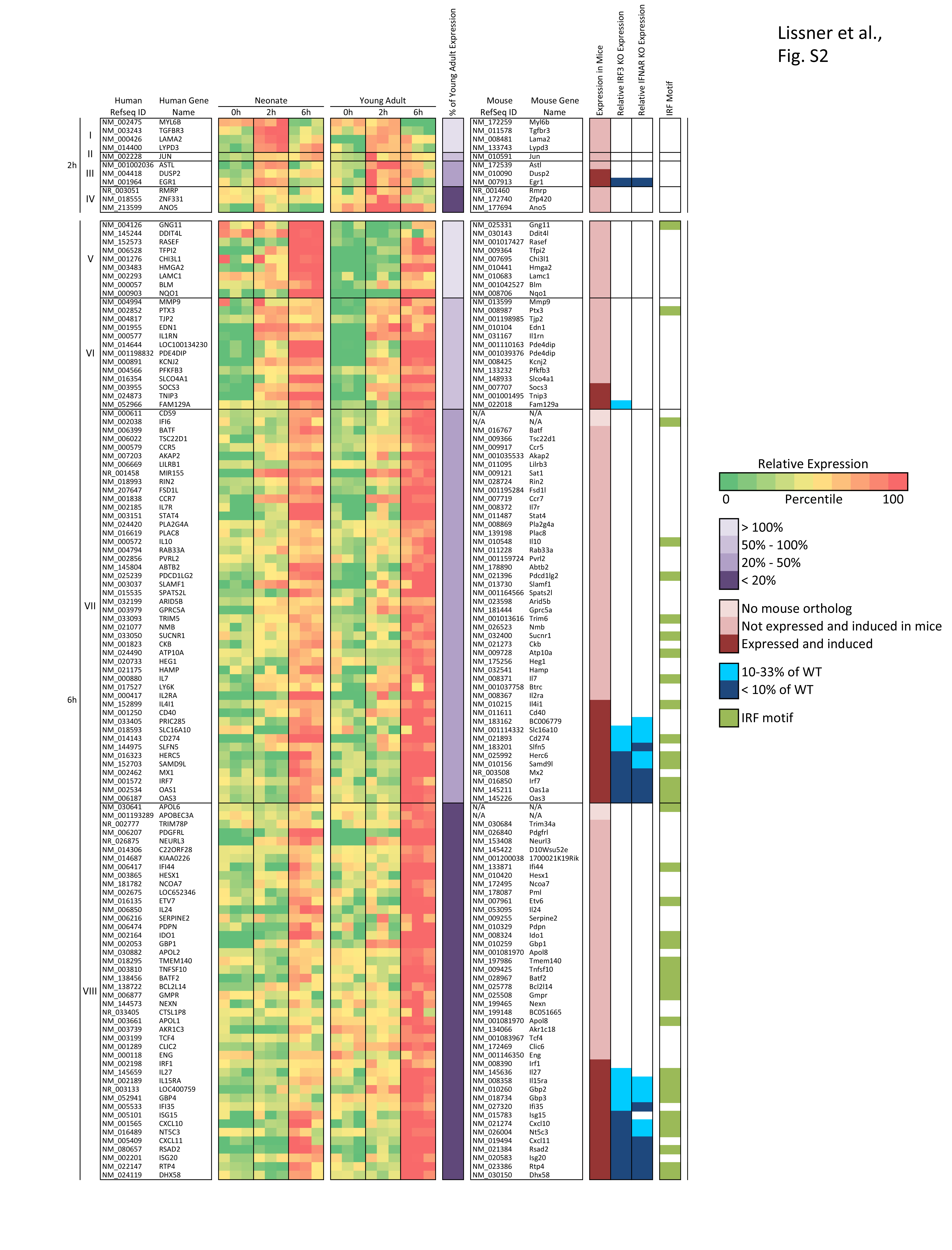

Supplement: S2 Fig — An expanded version of Fig 6A is shown, which includes the identities of the Lm-induced genes that are differentially expressed in cord blood and young adult monocytes. RefSeq IDs and gene names are shown for human genes and their mouse orthologs. (TIF) [file pone.0132061.s002.tif]

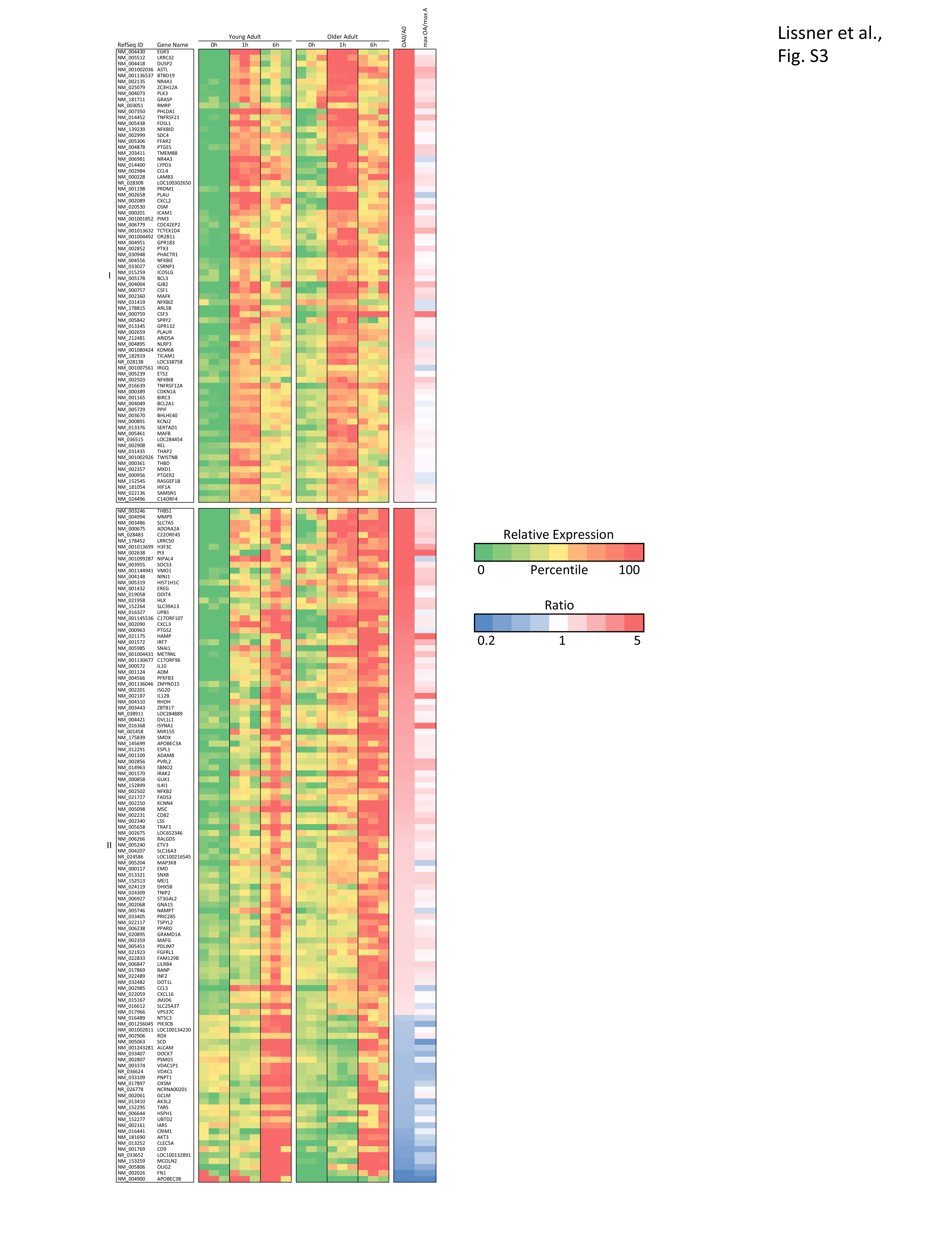

Supplement: S3 Fig — An expanded version of Fig 7A is shown, which includes the identities of LPS-induced genes that are differentially expressed in unstimulated young and older adult monocytes. Human RefSeq IDs and gene names are shown. (TIF) [file pone.0132061.s003.tif]
